# Higher spatial resolution improves the interpretation of the extent of ventricular trabeculation

**DOI:** 10.1111/joa.13559

**Published:** 2021-09-26

**Authors:** Hanne C. E. Riekerk, Bram F. Coolen, Gustav J. Strijkers, Allard C. van der Wal, Steffen E. Petersen, Mary N. Sheppard, Roelof‐Jan Oostra, Vincent M. Christoffels, Bjarke Jensen

**Affiliations:** ^1^ Department of Medical Biology Amsterdam Cardiovascular Sciences University of Amsterdam Amsterdam UMC Amsterdam The Netherlands; ^2^ Department of Biomedical Engineering and Physics Amsterdam Cardiovascular Sciences University of Amsterdam Amsterdam UMC Amsterdam The Netherlands; ^3^ Department of Pathology Amsterdam Cardiovascular Sciences University of Amsterdam Amsterdam UMC Amsterdam The Netherlands; ^4^ William Harvey Research Institute NIHR Barts Biomedical Research Centre Queen Mary University of London London UK; ^5^ Barts Heart Centre St Bartholomew’s Hospital Barts Health NHS Trust London UK; ^6^ Department of Cardiovascular Pathology Cardiology Clinical Academic Group, Molecular and Clinical Sciences Research Institute St George’s University of London London UK

**Keywords:** cardiomyopathy, heart, magnetic resonance imaging, noncompaction

## Abstract

The ventricular walls of the human heart comprise an outer compact layer and an inner trabecular layer. In the context of an increased pre‐test probability, diagnosis left ventricular noncompaction cardiomyopathy is given when the left ventricle is excessively trabeculated in volume (trabecular vol >25% of total LV wall volume) or thickness (trabecular/compact (T/C) >2.3). Here, we investigated whether higher spatial resolution affects the detection of trabeculation and thus the assessment of normal and excessively trabeculated wall morphology. First, we screened left ventricles in 1112 post‐natal autopsy hearts. We identified five excessively trabeculated hearts and this low prevalence of excessive trabeculation is in agreement with pathology reports but contrasts the prevalence of approximately 10% of the population found by *in vivo* non‐invasive imaging. Using macroscopy, histology and low‐ and high‐resolution MRI, the five excessively trabeculated hearts were compared with six normal hearts and seven abnormally trabeculated and excessive trabeculation‐negative hearts. Some abnormally trabeculated hearts could be considered excessively trabeculated macroscopically because of a trabecular outflow or an excessive number of trabeculations, but they were excessive trabeculation‐negative when assessed with MRI‐based measurements (T/C <2.3 and vol <25%). The number of detected trabeculations and T/C ratio were positively correlated with higher spatial resolution. Using measurements on high resolution MRI and with histological validation, we could not replicate the correlation between trabeculations of the left and right ventricle that has been previously reported. In conclusion, higher spatial resolution may affect the sensitivity of diagnostic measurements and in addition could allow for novel measurements such as counting of trabeculations.

## Graphical abstract

Jensen et al. show that higher spatial resolution may affect the sensitivity of diagnostic measurements of noncompaction and in addition could allow for novel measurements such as counting of trabeculations.

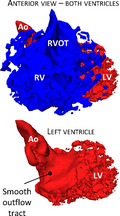

## 1 INTRODUCTION

Trabeculations line the luminal side of the walls of the human cardiac ventricles and compact wall comprises the epicardial side (Greenbaum et al., 1981; Streeter Jr, 1979). Much attention is given to trabeculations because they lead to the diagnosis of noncompaction cardiomyopathy when they are excessive and when there is a clinical suspicion or increased pre‐test probability (Arbustini et al., 2016; Jacquier et al., 2010; Petersen et al., 2018; Thuny et al., 2010). Noncompaction is the hypothesized aetiology defined as a failure of compaction (Chin et al., 1990), where compaction itself is a process originally described in chicken whereby embryonic trabeculation coalesce to become compact wall (Rychterova, 1971). To date, however, no hard evidence exists for compaction in human (Faber et al., 2021b). Instead, trabeculations can be measured to grow throughout gestation (Blausen et al., 1990; Faber et al., 2021a, 2021c) rather than exhibiting the decrease in volume that would be expected if they coalesced into compact wall (Faber et al., 2021b). In this study, therefore, we prefer the term ‘excessive trabeculation’ as introduced by (Anderson et al., 2017). It describes the setting of a few large trabeculations, or hypertrabeculation (Finsterer et al., 2017), and, or, the setting of a great number trabeculations, or so‐called ‘noncompaction’, while being neutral to the aetiology, or aetiologies, behind excessive trabeculation (Anderson et al., 2017).

It was originally in *post*‐*mortem* hearts that a setting of excessive trabeculation was first described. The setting was considered to be rare and characterized by a large amounts of tiny embryonic‐like trabeculations as revealed by histology (Finsterer & Zarrouk‐Mahjoub, 2013). Now, excessive trabeculation is diagnosed almost exclusively on the basis of non‐invasive imaging such as echocardiography and MRI (Amzulescu et al., 2015; Andreini et al., 2016; Grigoratos et al., 2019; Taylor & Nutting, 2021; Towbin & Jefferies, 2017). Commonly used diagnostic criteria use measurements of the relative thicknesses and masses of trabecular and compact layers (D'Silva & Jensen, 2020; Oechslin et al., 2000; Petersen et al., 2005; Di Toro et al., 2021). Concomitantly, diagnosis has become much more prevalent (D'Silva et al., 2020; Weir‐McCall et al., 2016). Images from echocardiography and MRI have a substantially lower spatial resolution than microscopy of histological sections, and changes in imaging resolution are thought to drive some of the increase in prevalence (Hussein et al., 2015).

When structures have a size smaller than the lower limit of the spatial resolution of the images they are assessed from, the number and extent of such structures may be underestimated. An example of this principle is the recent upward adjustment of the measured number of trees (Brandt et al., 2020). The foundational descriptions of excessively trabeculated ventricles showed individual trabeculations, or trabeculae carneae, with a width of a fraction of a millimeter (Burke et al., 2005; Dusek et al., 1975; Feldt et al., 1969; Freedom et al., 2005; Grant & Regnier, 1926; Ursell, 2013). Cardiac clinical MRI usually deploys a resolution of, for example, 1.8 × 1.8 × 10mm (Xia et al., 2021). In the absence of direct comparisons, the relation between what non‐invasive imaging captures and what the pathologist sees macroscopically and histologically remains unclear (Stollberger & Finsterer, 2021). In addition, if compaction occurs, it is thought to reduce the number of trabeculations, but these are too numerous be counted precisely by the unaided eye (Gerger et al., 2013). Counting of trabeculations will then have to be done on images, but the number of identifiable trabeculations may be limited by the spatial resolution.

The aim of this study was to test the hypothesis that spatial resolution impacts on the measurement of ventricular trabeculation. To do so, we use *post*‐*mortem* hearts to obtain transmural histology and MR‐based images with a resolution that exceeds that of usual clinical non‐invasive imaging investigations. We reasoned that any impact of spatial resolution would be more readily discovered if the investigated hearts exhibited a broad range in the extent of trabeculation. Thus, we first screened pathology archives for hearts that were excessively or abnormally trabeculated and compared these with hearts without abnormalities. Incidentally, this approach allowed for an assessment of the prevalence of hearts that fulfill structural criteria for excessive trabeculation. We hypothesize that a greater number of trabeculations may be detected when the left ventricular wall is assessed at higher spatial resolutions. We also assess whether the thickness of the trabecular layer relative to that of the compact layer (T/C ratio) is dependent on spatial resolution. Wall ratios are rarely measured for the right ventricle (RV) because the width of its compact wall is at the limits of the spatial resolution of non‐invasive imaging (Nucifora et al., 2014; Rao et al., 2020; Stollberger et al., 2015). In this study we could measure the thickness of the RV compact wall as well, and this enables us to test whether the mass and proportion of trabecular muscle of the RV and of the LV are correlated.

## 2 MATERIALS AND METHODS

**FIGURE 1:**
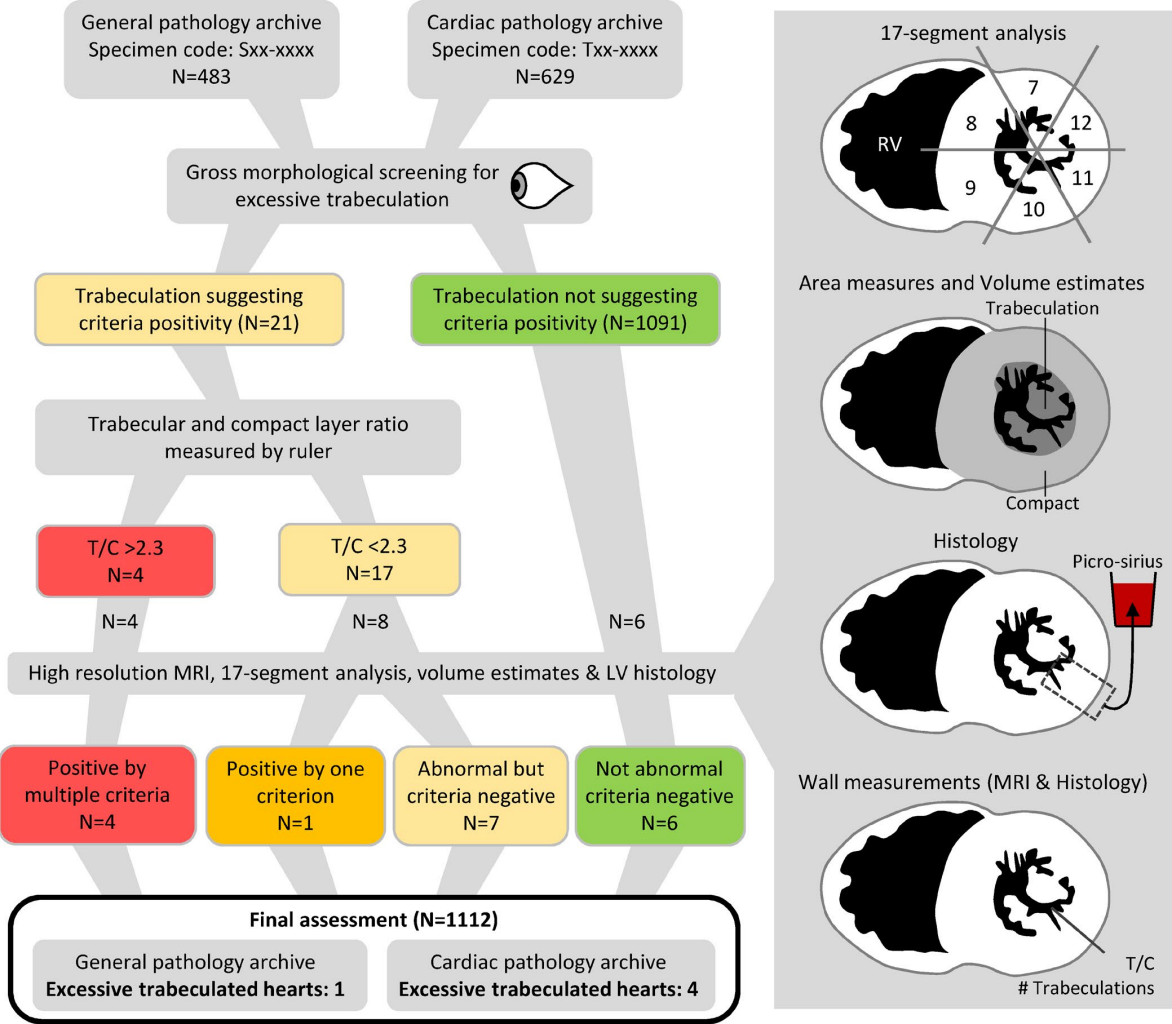
The selection of hearts for detailed investigation. T/C, trabecular layer thickness relative to the compact layer thickness [Colour figure can be viewed at wileyonlinelibrary.com]

Our study was retrospective and anonymized and therefore did not require informed consent in compliance with institutional ethical guidelines and the principles outlined in the Declaration of Helsinki. The study design and work flow are illustrated in Figure 1. The data that support the findings of this study are available from the corresponding author upon reasonable request.

### 2.1 Specimen archives and screening

**FIGURE 2:**
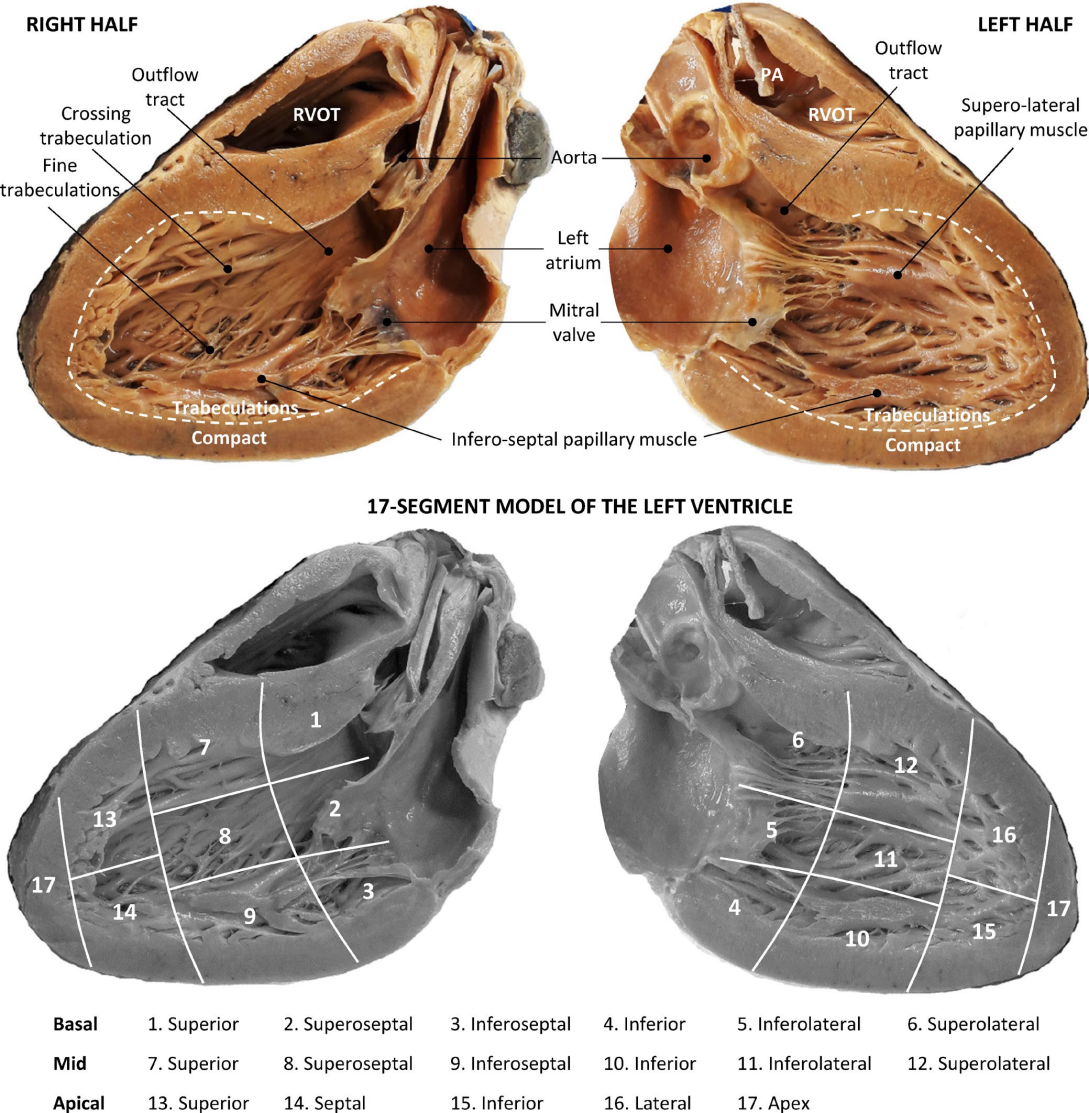
Normal anatomy of the left ventricle of the human heart in the attitudinal appropriate orientation. This heart was cut in so‐called 3 chamber‐view, showing the right half on the top‐left and the left half on the top‐right. Note that almost the entire ventricular wall has trabeculation, only the outflow tract has a smooth wall. There are fine trabeculation between the infero‐septal papillary muscle and the septal surface (left‐hand image). A few trabeculations cross over from the anterior wall to the septal surface (see Crossing trabeculation). The bottom images show the approximate manner of dividing the left ventricle into the 17 segments of the 17‐segment model (compared with the original nomenclature of the 17 segments (Cerqueira et al., 2002), ‘anterior’ has been substituted by the attitudinal correct ‘superior’ (Partridge & Anderson, 2009)). PA, pulmonary artery; RVOT, right ventricular outflow tract [Colour figure can be viewed at wileyonlinelibrary.com]

The hearts were archived between 1972 and 1998, at a time when there was little awareness of excessive trabeculation (D'Silva and Jensen, 2020). A previous study on 474 autopsy hearts (Boyd et al., 1987) showed no effect of sex and age on the prevalence of prominent LV trabeculations. We therefore chose to screen the hearts while blind to sex and age. Many specimens had no archived medical history and we could not differentiate between excessive trabeculation of genetic, pathological, physiological or sporadic origin (van Waning et al., 2019). Our principal approach was to set aside hearts with LVs that were highly trabeculated and then investigate these macroscopically, histologically and with MRI. These hearts were analyzed by sequential segmental analysis for acquired and congenital malformations (Anderson et al., 1984), the left ventricular wall was assessed by the 17‐segment model of the LV used for clinical imaging (Cerqueira et al., 2002) (with ‘anterior’ substituted by the attitudinal correct ‘superior’ (Partridge & Anderson, 2009)) and we used the attitudinal appropriate nomenclature for structures and positions (Anderson et al., 2013). Figure 2 shows a structurally normal heart and gives an overview of the nomenclature for structures and positions used in this study (Anderson et al., 2013; Cerqueira et al., 2002; Partridge & Anderson, 2009). It also illustrates that the trabecular and compact layers are approximately equally thick when viewed with the unaided eye and the T/C ratio is well below the threshold for excessive trabeculation (T/C >2.3 (Petersen et al., 2005)).

The hearts were either from our in‐house pathology department (S‐archive, coded as S(year)‐(specimen number), *N* = 483) or they were sent to our institution for assessment (T‐archive, coded as T(year)‐(specimen number), *N* = 629). The only patient information we could retrieve for all cases was year of dying (all within the period of 1972 and 1998), age at death and sex. The cause of death was not known for all cases. Regions with signs of myocardial infarction, including regional thinning of the wall and scarring, were disregarded in the assessment of trabeculations and wall thicknesses. The LVs were mostly exposed either by a series of short‐axis slices from the apex to approximately the mid‐height of the ventricle, resulting in 4–5 slices, or by a long‐axis cut to the anterior wall and the lateral wall. In most instances, the trabecular and compact layer had an approximately similar thickness (Figure 2), and the thicknesses were not measured with a ruler. Only the hearts in which the LV was perceived as having an abnormally high T/C value, in that it presented with an excessive number of trabeculations or with prominent trabeculation were set aside. These hearts were then surveyed along the cut planes of the autopsy for maximum T/C with a ruler and further analyzed by sequential segmental analysis for overt acquired and congenital malformations (Anderson et al., 1984). Hearts that were excessively trabeculated were investigated for evaluation of atherosclerotic coronary artery disease by making multiple transverse cuts along the course of the main epicardial arteries (Basso et al., 2017).

### 2.2 Magnetic resonance imaging and image analysis

For control hearts, we selected six hearts that were normal by external appearance and which had an unopened LV, and we were therefore blind to the state of the LV trabeculation. Later, we found their LV walls were well below the threshold values for excessive trabeculation and these hearts were then considered normal. As negative control hearts, we selected hearts with abnormally trabeculated LVs that had T/C less than 2.3 when measured macroscopically (one was later seen to meet threshold values for excessive trabeculation). Finally, as true cases, we selected three hearts in which the LV had at least one segment with a T/C greater than 2.3 when measured macroscopically (these were all excessively trabeculated by MRI‐based measurements). One heart that was excessively trabeculated macroscopically (S96‐232.0) and was not imaged with MRI because it could not be re‐assembled to anything close to the intact morphology due to it being cut in thin slices and its highly pliable walls.

Before MRI, hearts were rinsed in running tap water for several days. We reassembled the cut hearts to the best of our ability and wrapped them in gauze before submerging them in individual buckets filled with tap water. The hearts were scanned on a 3T Ingenia clinical MRI scanner (Philips, Best, the Netherlands) using a standard 16‐channel head coil. A 3D T1‐weighted gradient‐echo sequence was used in order to suppress the fluid for optimal myocardium/lumen contrast. Specific sequence parameters were: TR/TE = 5.7/2.4 ms, flip angle = 20 degrees, resolution = 0.5 × 0.5 × 0.5 mm^3^, number of averages = 4, total scan time = 12 min. After scanning, the hearts were transferred to preservative and tissue blocks were collected later for histology.

To orient the MRI‐generated image stacks to the three conventional planes (short‐axis/transverse, 2 chamber‐view, 4 chamber‐view), we used Amira (Ver. 6.5.0 or 2019.3, Thermo Fisher Scientific). Briefly, the module ‘Volume rendering’ was used to get a preliminary 3D model and we then used ‘Transform editor’ to rotate the rendered volume to the three conventional planes. To save this transformation, we used ‘Resample Transformed Image’ (settings: Interpolation, Lanczos; Mode, extended; Preserve, Voxel size). Next, we used the short‐axis images to identify images for the 17‐segment model analyses. To divide the LV into the basal, mid, and apical parts of the 17‐segment model, we identified landmark images containing the basal most part of the LV, the tips of the papillary muscles, the base of the papillary muscles, and the last part of the apex. The images for analyses of basal, mid, and apical segments were then found midway between two landmark images.

### 2.3 Histology

We cut from 16 hearts LV and RV transmural tissue blocks, and from one heart we cut a trans‐septal block from its abnormally trabeculated outflow tract (T74‐385). The tissue blocks came from hearts that were normal (five hearts), abnormally trabeculated but not excessively trabeculated (six hearts) or excessively trabeculated (five hearts). These samples were used to characterize the LV histologically, to count the number of trabeculation in that location, and to measure T/C (in comparison to MRI, see below). The tissue blocks were imbedded in paraplast, sectioned at 10 µm thickness on a microtome, and stained in saturated picro‐sirius red solution followed by 2 min differentiation in 0.01 M HCl (myocardium is orange, collagen is red). Each histological section was imaged at a resolution of 258 pixels/mm with approximately 10 photos per section which were stitched to a single image (jpeg) in Photoshop CS6 (ver. 13.0.1, Adobe) using the ‘Photomerge’ function (settings: Layout, Auto).

### 2.4 Statistical analyses

We used Chi‐square to test whether there was a difference in the prevalence of excessive trabeculation in the two archives. Short‐axis MRI images were analyzed in Amira (v6.5.0 or 2019.3, Thermo Fisher Scientific) for the 17‐segment analyses, to measure T/C, and to measure the volume of trabecular and compact wall by the Cavalieri principle (Gundersen et al., 1988), or Simpson's rule. A minimum of 10 equidistant images were used per heart. Papillary muscle and other ventricular trabecular muscle develop from embryonic trabecular muscle (Anderson et al., 2017; Miquerol et al., 2010) and given this common origin we included papillary muscle in all measurements. A left ventricular trabecular volume in excess of 25% of the total LV wall was taken to indicate excessive trabeculation (Grothoff et al., 2012). Intertrabecular recesses (Jacquier et al., 2010) were not included in the trabecular area measurements. For statistical analyses, we linearly correlated the trabecular proportion of the LV wall to the T/C per segment (except the apex, segment 17) using Pearson correlations in which the threshold for significance was (Bonferroni) corrected for the number of tests (significance at *p* < 0.05/16). Lastly, we used images from histology, high‐resolution MRI, and low‐resolution MRI to investigate whether spatial resolution affected the T/C and the number of trabeculations that could be counted, using two‐factor (sample and spatial resolution) ANOVA in which *p* < 0.05 was considered to be statistically significant. Statistics were computed in Excel (version 16.16.27). The low‐resolution images were made to approximate the resolution of typical clinical imaging by resampling the image stacks from the original resolution of 0.5 × 0.5 × 0.5 mm^3^ to 1.5 × 1.5 × 8.0 mm^3^ using the ‘Crop editor’ function in Amira. Images were imported to ImageJ (v1.56, NIH) and we counted the number of trabeculations along trajectories from epicardium to endocardium and measured the thickness of the trabecular (NC) and compact wall (C).

## 3 RESULTS

### 3.1 Selection of hearts

We screened 1112 hearts in which the trabeculation of the entire LV was assessed macroscopically, including an assessment of the relative thicknesses of the trabecular and compact layer along all cut surfaces. Very few of the screened hearts appeared excessively trabeculated and ultimately only five were found to be quantitatively excessively trabeculated (see Figure 1 and below). Four of the five excessively trabeculated hearts came from the T archive (*N* = 629), one heart came from the S archive (*N* = 483), but the prevalence of excessive trabeculation was not significantly different between the two archives (*p* = 0.295). In addition to the five excessively trabeculated hearts, we selected 13 hearts on the basis of their normally (*n* = 7) or abnormally trabeculated (*n* = 6) appearance (Figure 1). The 18 hearts in total were analyzed in depth.

### 3.2 Validation of distinction between trabecular and compact myocardium

**FIGURE 3:**
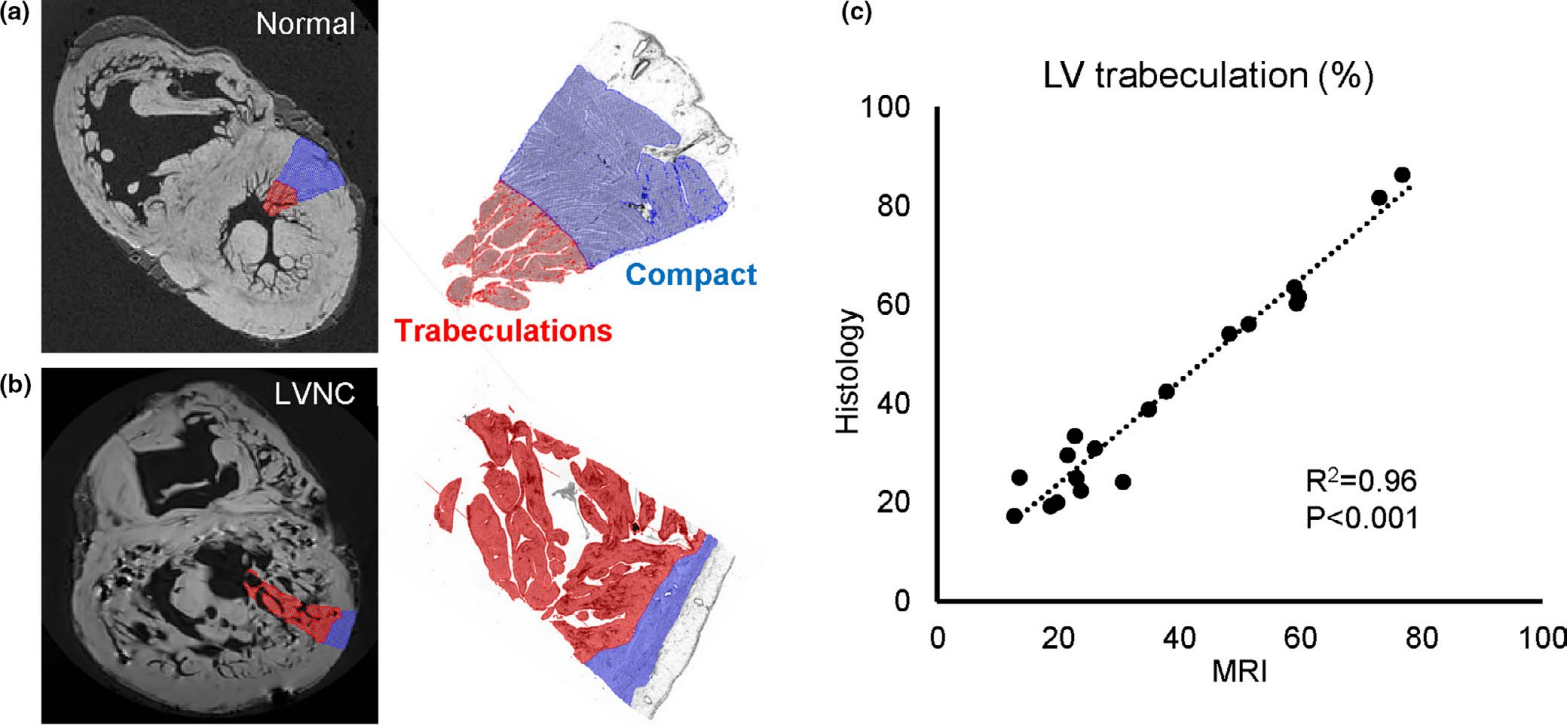
Validation of left ventricle labelling on high‐resolution MRI with histology (trabecular muscle in red, compact muscle in blue). (a) Labelling of a normal left ventricular wall. (b) Labelling of a very excessively trabeculated left ventricular wall. (c) Significant correlation of proportion of trabecular muscle on the basis of MRI and histology of 19 regions from 16 hearts [Colour figure can be viewed at wileyonlinelibrary.com]

**FIGURE 4:**
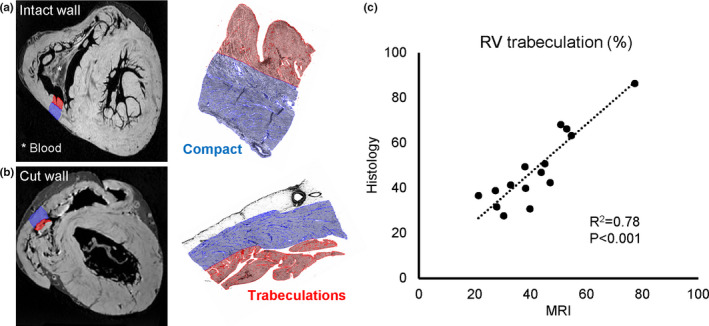
Validation of right ventricle labelling on high‐resolution MRI with histology (trabecular muscle in red, compact muscle in blue). (a) Labelling of a normal right ventricular wall. (b) Labelling of a difficult right ventricular wall that has been perturbed by cuts from the autopsy and by displacement of the wall. (c) Significant correlation of proportion of trabecular muscle on the basis of MRI and histology of 16 regions from 16 hearts [Colour figure can be viewed at wileyonlinelibrary.com]

One diagnostic criterion used here is that excessive trabeculation can be assigned if the labelled trabecular volume exceeds 25% of total LV volume (Grothoff et al., 2012; Macaione et al., 2021). Therefore, we first tested whether our labelling of trabecular and compact myocardium on the basis of high‐resolution MRI corresponded to the labelling based on histology. We deliberately selected transmural samples with great variation in degree of trabeculation and complexity of appearance. Figures 3, 4 show that labellings on the basis of MRI and histology were strongly and highly significantly correlated for both LV and RV (LV, *R* ^2^ = 0.96, *p* < 0.001; RV, *R* ^2^ = 0.78, *p* < 0.001).

### 3.3 Analysis of excessively trabeculated hearts

Of all hearts, the five excessively trabeculated hearts had the greatest proportion of trabeculation which in every case exceeded 25% of total LV wall volume (Table 1). In addition, they also had the greatest T/C ratios (Table 1). Two observers (HCER, BJ) labelled the proportion of left ventricular trabecular muscle and both found that the measured volume percentages were positively and highly significantly correlated (single factor ANOVA, *p* < 0.001) and not different (single factor ANOVA, *p* = 0.769). The anatomical findings and measurements of each case are summarized in Table 2. Common to all five cases were male sex and some degree of LV dilation. The outflow tract was not excessively trabeculated as trabeculations were absent. We correlated the percentage of LV trabecular volume to the T/C score of each segment (1–16, excluding the apex). Only the apical segments (13–16) had a significant correlation (Bonferroni‐corrected threshold, *p* = 0.05/16), in agreement with the observation that excessive trabeculation is most frequently found in the apical segments (Petersen et al. 2018).

**TABLE 1:** Summary of MRI‐based comparisons of normal hearts, hearts that were abnormally trabeculated but not excessively trabeculated (Abnormal), and excessively trabeculated hearts (ET)

	Normal (*N* = 6)	Abnormal (*N* = 7)	ET (*N* = 4)	*p* value
T/C >2	0	3	4	
T/C >2.3	0	0	3	
Trabecular volume >25%	0	1	4	
Average T/C (Segments 1–16)	0.57 (0.11)	0.66 (0.16)	1.45 (0.91)	**0.016**
Summed T/C (Segments 1–16)	8.86 (1.65)	10.40 (2.61)	21.92 (13.82)	**0.021**
% LV trabecular volume	15.6 (1.7)	20.8 (3.2)	34.2 (4.8)	**<0.001**

Note

*p* values are of one‐way ANOVAs. Values in the last three rows are given as average (standard deviation).

**TABLE 2:** Key findings on the five excessively trabeculated hearts

Case	1	2	3	4	5
Age at death	74	80	52	43	31
Sex	male	male	male	male	male
Macroscopic assessment					
Max T/C	6,7	3,3	3	<2,3	3,3
Severe CAD	No	yes	no	no	no
LV cavity	Dilated	mildly dilated	extremely dilated	mildly dilated	dilated
Trabecular OFT	No	no	no	no	no
MRI assessment					
Max T/C	8,5	5,2	no MRI	3	<2,3
Segments with T/C >2.3	7	3	no MRI	3	0
Trabecular volume (%)	40,9	29,5	no MRI	32,5	34
Histology					
Mural fibrosis	Patchy	patchy	patchy	extensive	patchy

**FIGURE 5:**
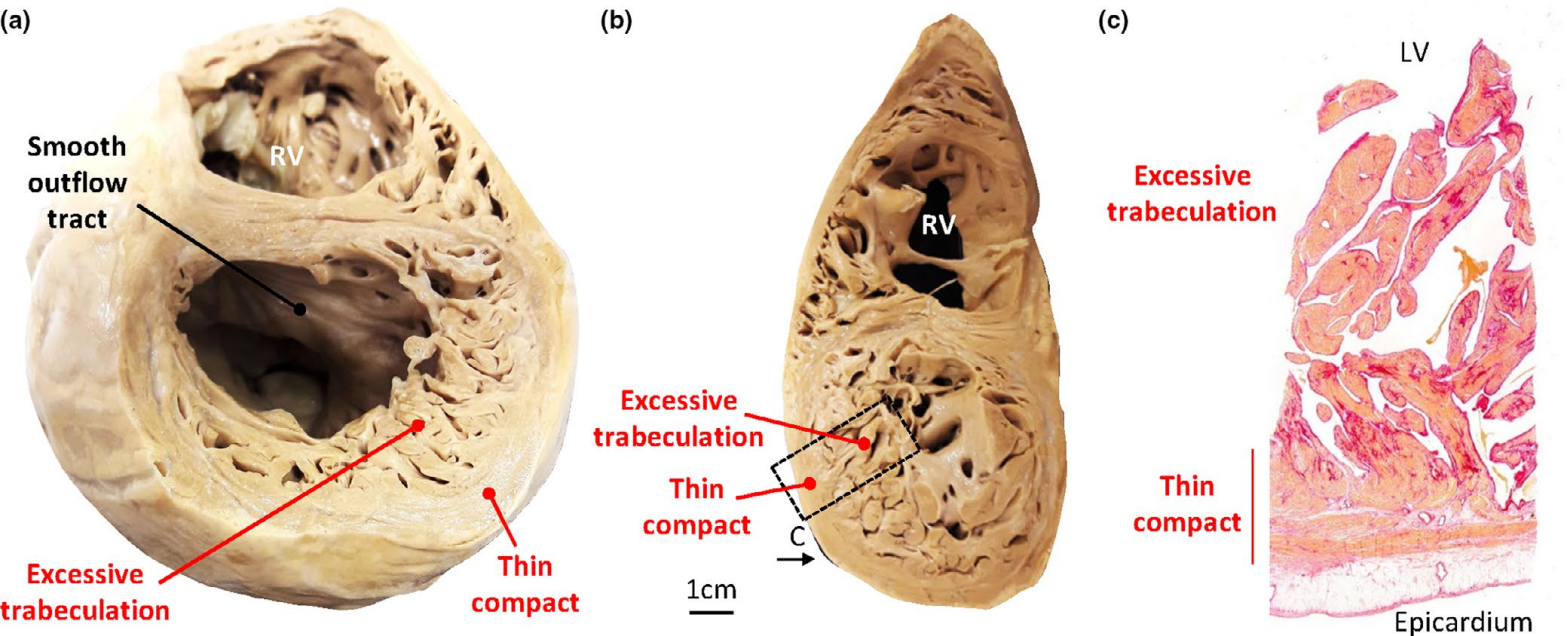
Excessive trabeculation Case 1. (a–b) Heart T91‐14080 with a dilated left (and right) ventricle, thin compact wall and trabeculations that are extremely excessive in number and prominence, both at mid‐height (a) and apically (b). The outflow tract is without trabeculations (a). (c) Histology showing an extremely thin compact wall regionally and patchy fibrosis in the trabeculations [Colour figure can be viewed at wileyonlinelibrary.com]

**FIGURE 6:**
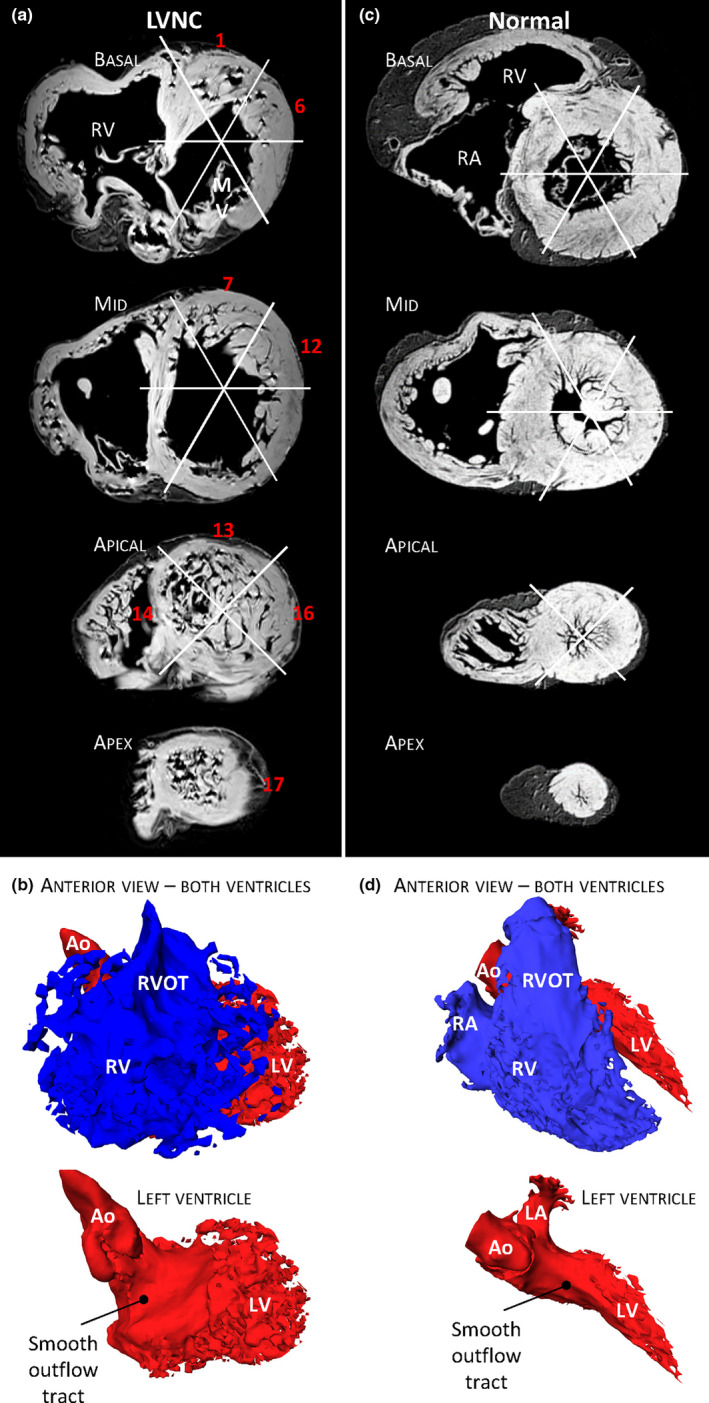
Excessive trabeculation Case 1 compared with a normal heart. (a) MRI images of Case 1 from which all 17 segments were assessed. Numbers in red indicate the segment number with a T/C greater than 2.3 (excessively trabeculated (Petersen et al., 2005)). (b) Ventricular lumens cast in anterior view, showing a spongy appearance to the left ventricular lumen (LV) apically, laterally and basally. (c) MRI images of a normal heart, no segments had a T/C greater than 2.3. (d) Ventricular lumens cast in anterior view, showing a less spongy left ventricular lumen than in (b). Ao, aorta; LA, left atrium; Mv, mitral valve; RA, right atrium; RV, right ventricle; RVOT, right ventricular outflow tract [Colour figure can be viewed at wileyonlinelibrary.com]

**FIGURE 7:**
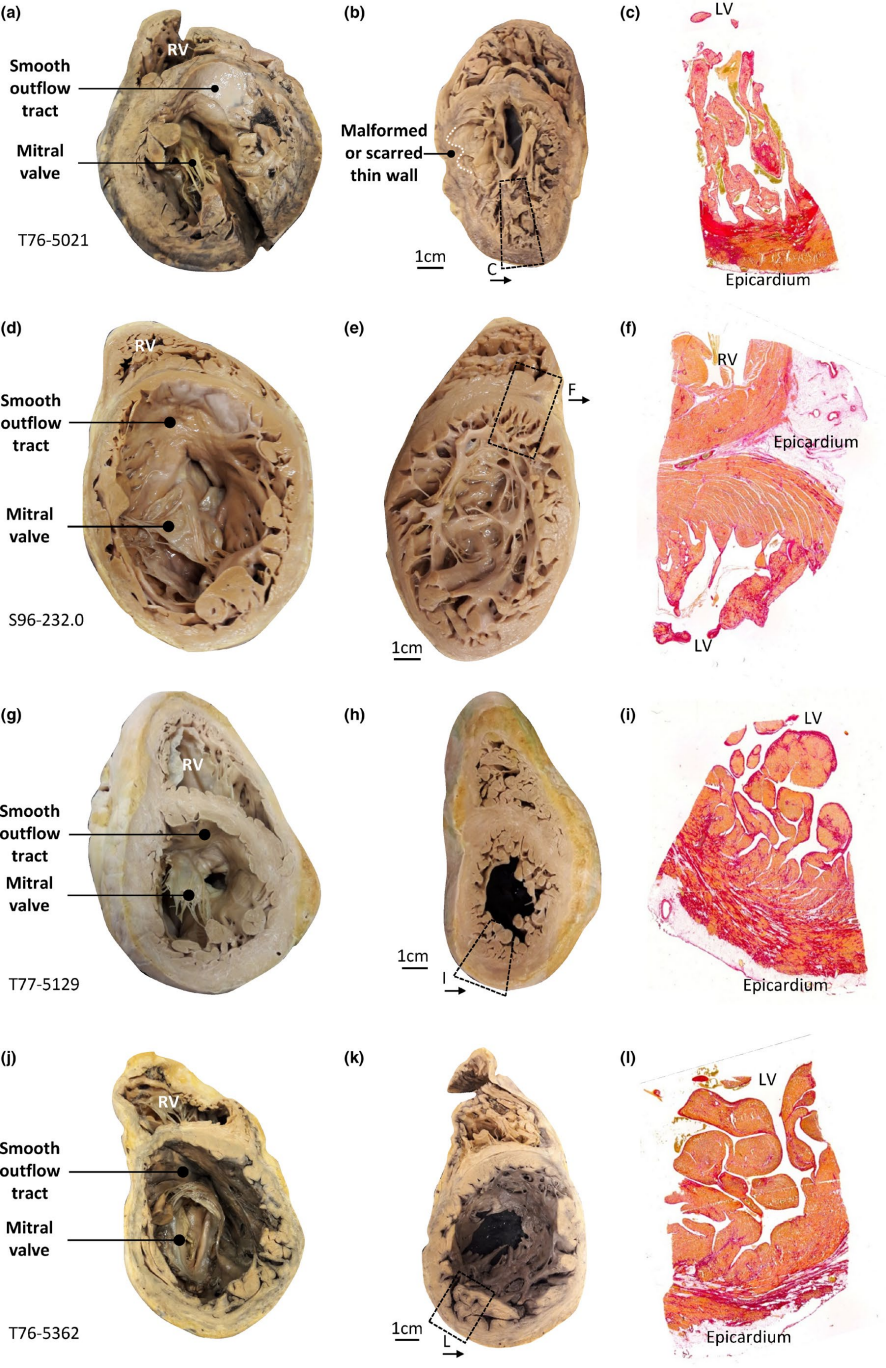
Excessive trabeculation Cases 2–5. (a‐c) Heart T76‐5021 showing a dilated left ventricle (a), with a thin compact wall (b), locally due to scarring. The trabeculations were very prominent relative to the thin compact wall, in part due to an excessive number of trabeculations (b). The outflow tract was smooth (a). Fibrosis was patchy in the trabeculations and compact wall (c). (d–f) Case 3 (Heart S96‐232.0) showing an extremely dilated left ventricle (d) with a thin compact wall (d–e). The trabeculations were prominent relative to the thin compact wall, but otherwise the trabecular layer was not thick, the number of trabeculations was not excessive and the outflow tract was smooth (d–f). Fibrosis was more pronounced in the trabeculation than in the compact wall (f). (g–i) Case 4 (Heart T77‐5129) showing a well‐developed compact wall in the ventricular base and a smooth outflow tract (g), whereas the ventricular wall in the apical region (h) showed an excessive number of trabeculations and there was substantial fibrosis in the sub‐endocardium of the trabeculations and in the compact wall (i). Macroscopically and by histology, T/C was less than 2.3, but on MRI we found three segments to have T/C greater than 2.3. (j–l) Case 5 (Heart T76‐5362) showing a dilated left ventricle (j) with a somewhat thin compact wall (j–k). The trabeculations were prominent relative to the thin compact wall, but the number of trabeculations was not excessive and the outflow tract was smooth (j–k). Fibrosis was more pronounced in the compact wall than in the trabeculation (l) [Colour figure can be viewed at wileyonlinelibrary.com]

The LV of Case 1, from a 74‐year‐old male, was the most excessively trabeculated of all screened LVs (Figure 5). The T/C was greater than 5 in three segments and the volume of the trabeculations exceeded 40% of the total left ventricular wall. Reconstructed ventricular lumens showed a dilated state of the ventricles (Figure 6). On histology, the compact wall had multiple small spots of scar tissue that never were transmural (Figure 5c). Case 2 came from an 80‐year‐old male. The greatest T/C on MRI was 5.2, which was confirmed on histology, and the trabeculation volume comprised almost 30% of the total left ventricular wall (Table 2, Figure 7a–c). Patches of fibrosis were found in the compact wall and trabeculations, and the superior part of the septum had old scarring presumably caused by ischemia. Coronary artery disease was evident from old occlusion of the anterior interventricular artery. The other excessively trabeculated hearts, Cases 3–5, were less extreme than Cases 1–2 (Table 2). They had at least one segment with T/C >2.3, either on MRI, macroscopy, histology, or all three. In Case 3 (52‐year‐old male), the cardiac mass was greater than 800 g at autopsy or approximately twice the expected mass. The LV was extremely dilated, the compact wall was thin, 0.4–0.5 cm, yielding a T/C of 2.5–3.0 at multiple locations (Figure 7d,e). Histology revealed substantial fibrosis in the left ventricular wall (Figure 7f). Because this heart was not possible to reassemble, it was not imaged with MRI. Case 4 came from a 43‐year‐old male and was first categorized as not excessively trabeculated by macroscopic measurements but later considered excessively trabeculated because of MRI‐based measurements. All T/C measurements were less than 2.3 macroscopically, but on MRI three segments had T/C greater than 2.3 and the trabecular volume comprised more than 25% of the LV wall volume (Table 2, Figure 7g,h). There was extensive fibrosis of the compact wall, less so in the trabeculations (Figure 7i). In Case 5 (31‐year‐old male), the compact wall was measured macroscopically to be thin, 0.6–0.8 cm, yielding T/Cs greater than 3 laterally (Table 2, Figure 7j,k). On MRI, no segments had T/C >2.3, but the trabecular layer comprised more than 25% of the LV wall, and this, together with the macroscopically measured T/Cs, led us to consider this heart to be excessively trabeculated. The compact wall had some fibrosis (Figure 7l).

### 3.4 Abnormally trabeculated left ventricles in diagnosis‐negative hearts

**FIGURE 8:**
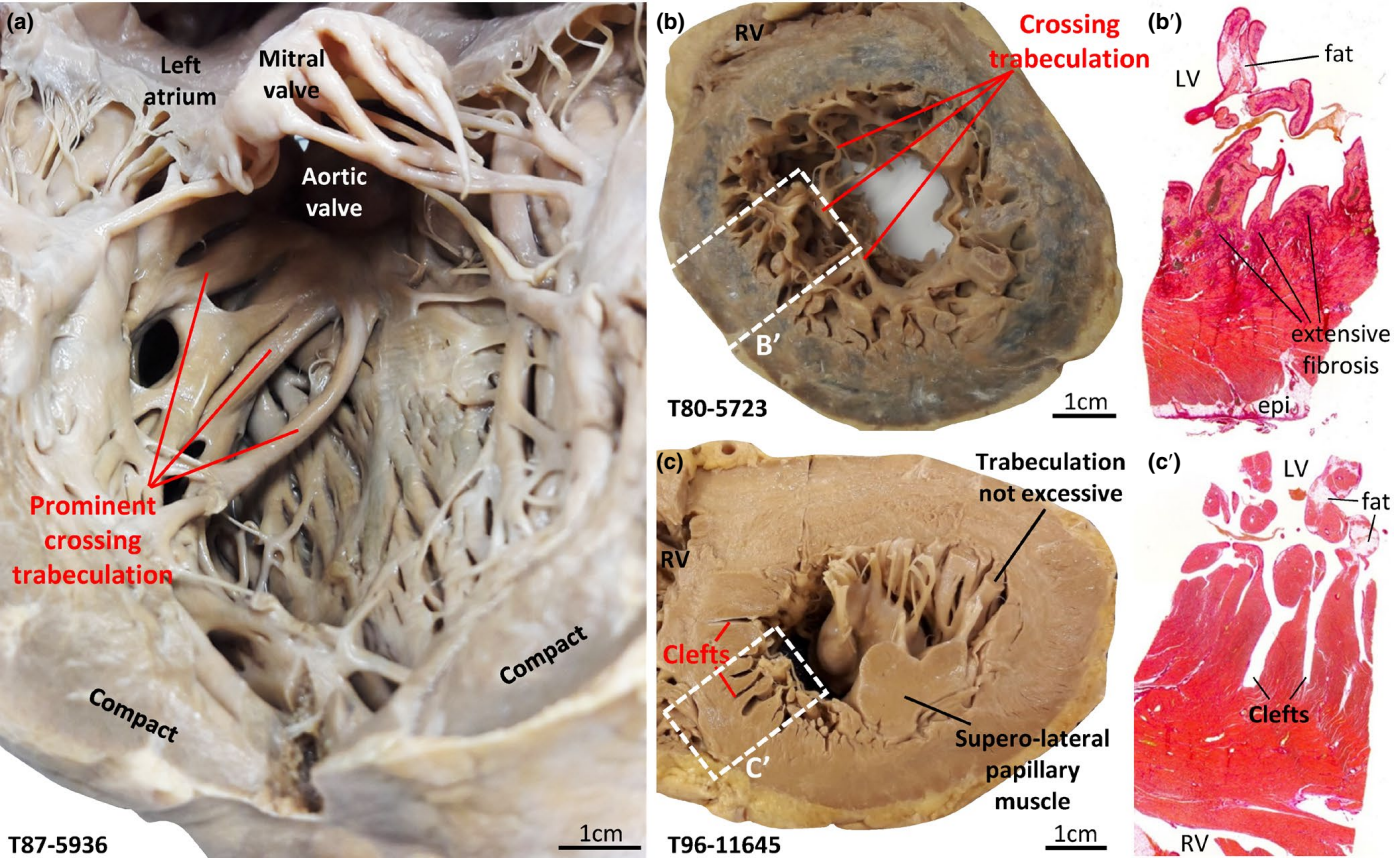
Abnormally trabeculated hearts. (a) Left ventricle with thick trabeculations that span much of the cavity from the apical part of the anterior wall to the outflow tract. (b) Short‐axis slice of the left ventricle, approximately at mid‐height of the ventricle, showing several fine trabeculations (possibly false tendons) crossing the cavity from the anterior wall to the septal surface. On histology (b’), extensive fibrosis was found in the LV wall and the crossing trabeculations contained substantial amounts of fat. (c) Short‐axis slice of left ventricle with deep clefts in luminal continuity with the left ventricular cavity within the compact ventricular septum and anterior wall (c’). epi, epicardium; LV, left ventricle; RV, right ventricle [Colour figure can be viewed at wileyonlinelibrary.com]

**FIGURE 9:**
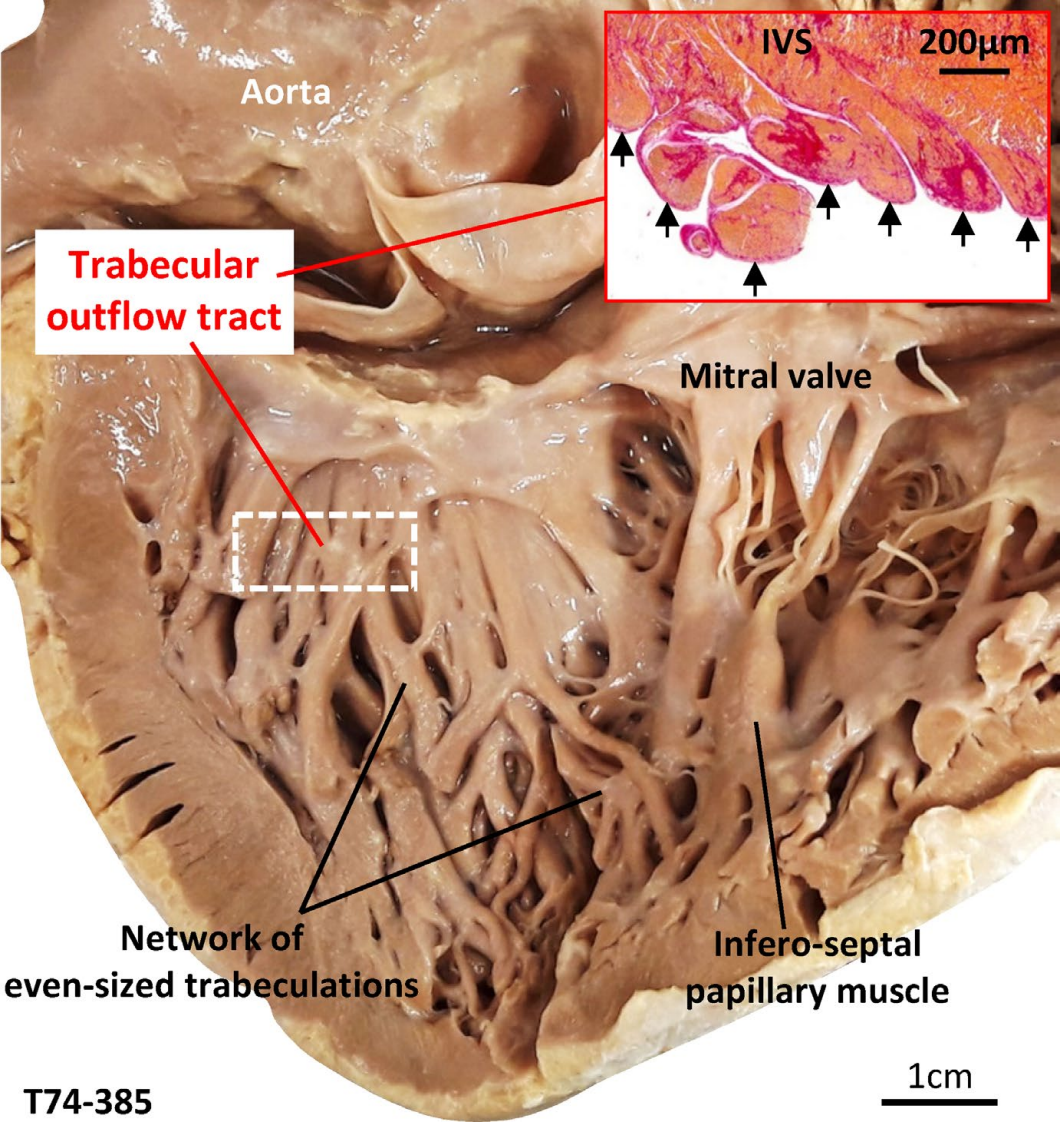
Left ventricle with unusual trabeculation. Left ventricle with numerous trabeculations in the outflow tract (indicated by arrows in insert) and most trabeculations were unusually even‐sized. IVS, interventricular septum [Colour figure can be viewed at wileyonlinelibrary.com]

**FIGURE 10:**
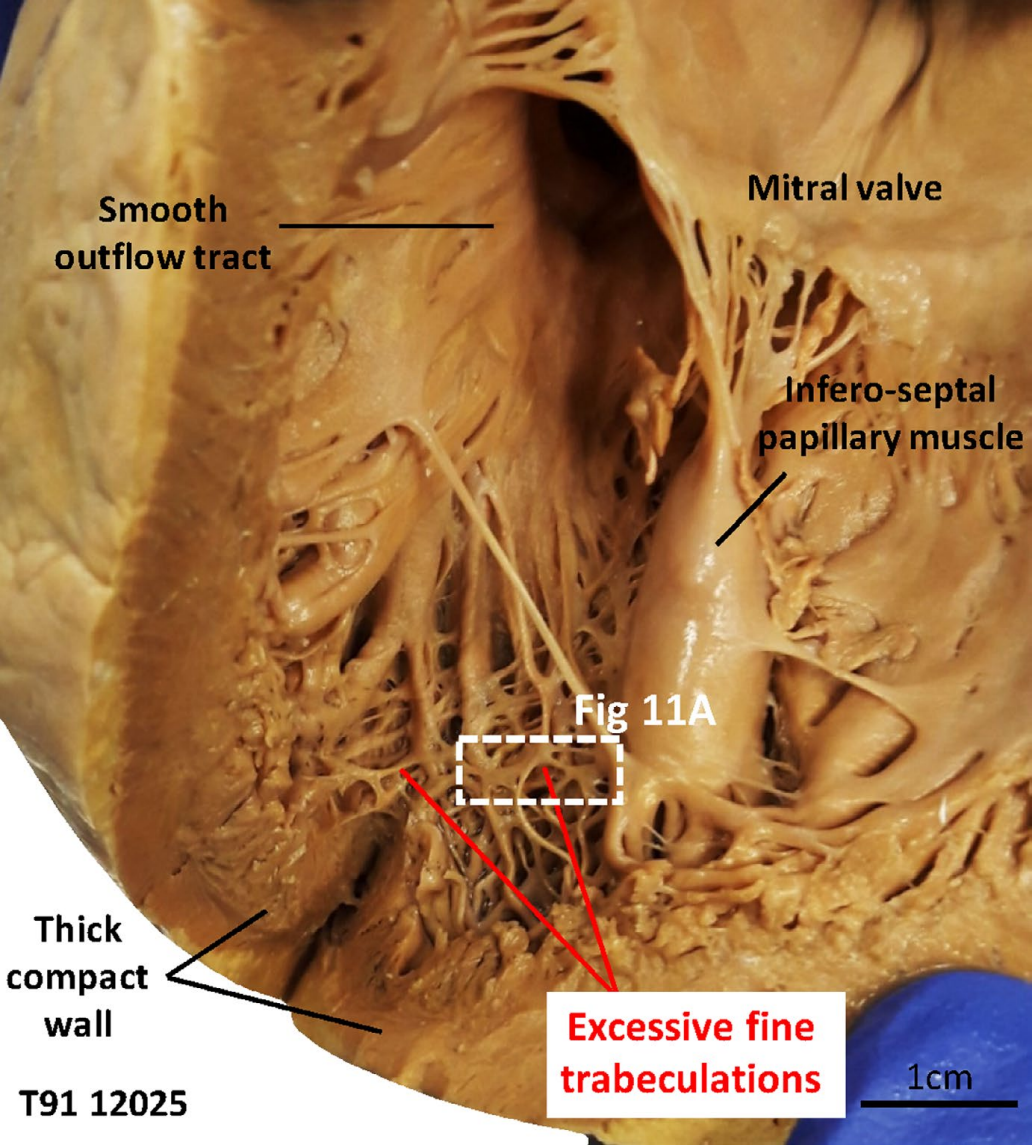
Left ventricle with excessive trabeculation and thick compact wall. Left ventricle with an excessive number of fine trabeculations together with a thick compact wall and a smooth outflow tract. The histology shown in Figure 11a comes from the region indicated by the dashed box [Colour figure can be viewed at wileyonlinelibrary.com]

We found several hearts that were abnormally trabeculated. The cases most ambiguous regarding diagnosis of excessive trabeculation are illustrated in Figures 8, 9, 10 and their measurements are summarized in Table 1. The measurements of T/C and volume of trabeculation relative to the total LV wall showed these hearts were more trabeculated than normal hearts, but less excessive than the excessively trabeculated hearts that fulfilled clinical diagnostic criteria (Table 1). The case illustrated in Figure 8a was the most ambiguous with regards to diagnosis. There were no overt signs of coronary artery disease. A few large trabeculations crossed the ventricular lumen (its peripheral part), which on echocardiography may warrant diagnosis (Jenni et al., 2001). Their prominence yielded T/C of 2 in segment 9 (MRI) and this measurement increased to 2.5 if we subtracted a layer of (likely) RV muscle from the septal compact wall. The T/C was less than 2 in all other segments. Although the trabecular layer volume comprised 25.9% of left ventricular wall volume, we deemed this case not to be excessively trabeculated because the compact wall was of normal thickness (Figure 8a). In the second example (Figure 8b), a few small trabeculations crossed from the anterior wall to the septal surface and these could likely be considered prominent false tendons (Luetmer et al., 1986). Histology revealed extensive fibrosis, both interstitial and patchy type, of the left ventricular wall and substantial fat in the crossing trabeculations (Figure 8b’). Besides the crossing trabeculations, the trabecular layer was not overtly abnormal, all 17 segments had a T/C of less than 2.3, and the trabeculations comprised 17.3% of total left ventricular wall. The third example is the only case we found with clefts in the ventricular septum and compact wall, in segment 9 (Figure 8c). Endocardium lined the clefts and if they were considered intertrabecular recesses, T/C reached the threshold value of 2.3. The trabeculations, however, comprised less than 25% of the left ventricular wall volume (21.3%). The fourth example was notable for having trabeculations in the outflow tract, which could be an expected outcome if compaction had failed, and that the trabeculations were unusually even‐sized (Figure 9). The trabeculations, however, comprised 20.1% of total left ventricular wall and the highest T/C was only 1.3. Lastly, the LV shown in Figure 10 is remarkable for its excessive network of fine trabeculation, which could be an expected outcome if compaction had failed. However, the compact wall was thick, the highest T/C was only 1.0, and the trabeculations comprised 17.2% of left ventricular wall volume. The diminutive size of the fine trabeculations appeared substantially below the spatial resolution of clinical MRI, and this suggested that clinical MRI may underestimate the number of trabeculation.

### 3.5 Number of trabeculations and T/C ratio

**FIGURE 11:**
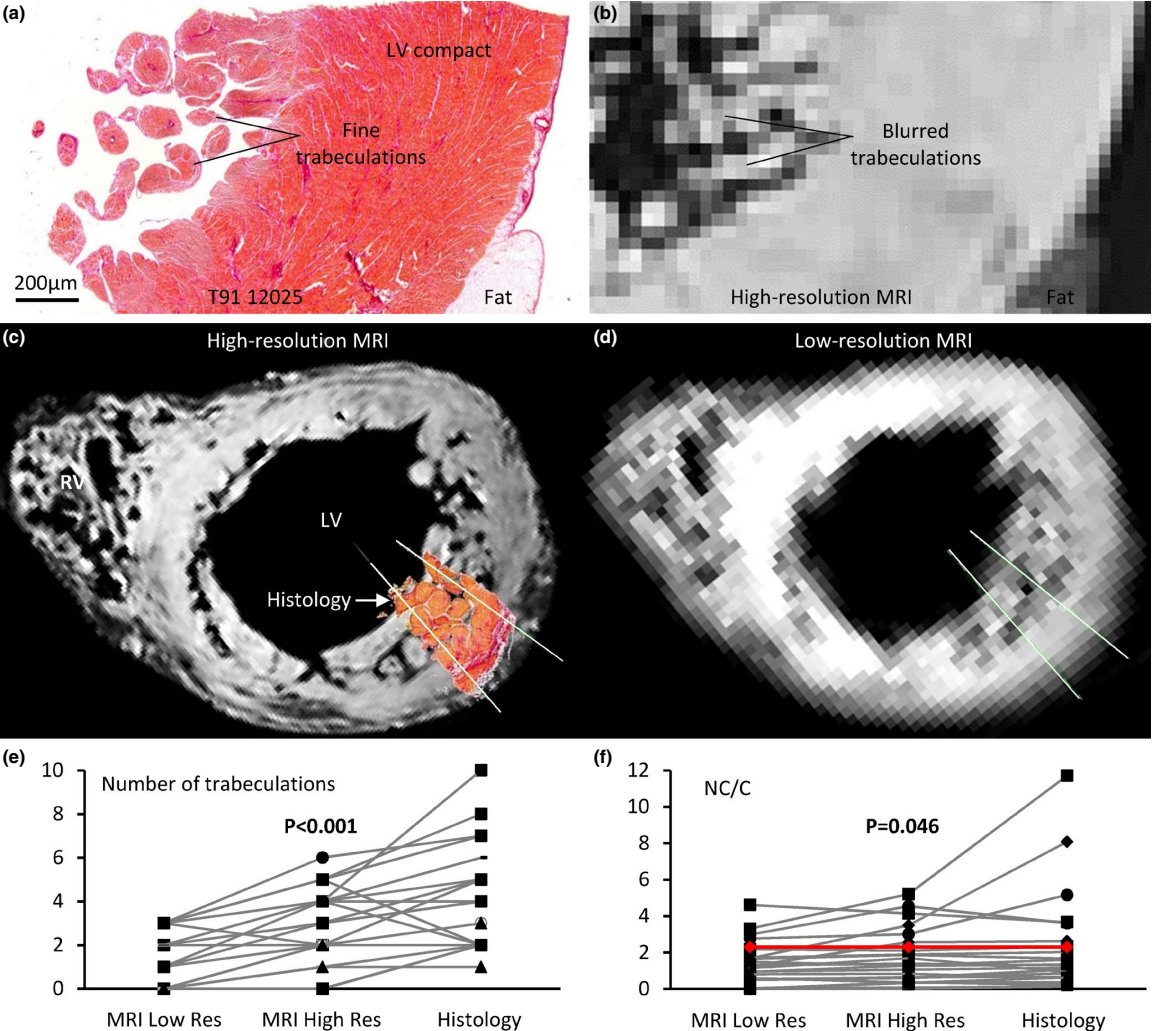
Left ventricular wall morphometrics. (a) Transmural histology of the LV wall of the heart shown in Figure 10, showing numerous fine trabeculations. (b) High resolution MRI (0.5 × 0.5 × 0.5 mm) of the same region as shown in (a), showing individual trabeculations are blurred together. (c) High‐resolution MRI short‐axis view of heart T76‐5362, onto which the histological section of Figure 7l has been placed in the position it was taken from. The white lines indicate the two trajectories along which the number of trabeculations was counted and T/C was measured. (d) Short‐axis image from the same position as shown in C from the image stack that was resampled to low spatial resolution (1.5 × 1.5 × 8 mm). (e) The number of detected trabeculations increased significantly with greater spatial resolution (two‐factor ANOVA). (f) A significantly greater T/C was measured with greater spatial resolution (two‐factor ANOVA). The red line indicates the threshold value (2.3) for excessive trabeculation by the criterion of (Petersen et al., 2005). The graphs of (e) and (f) are based on histology of 12 tissue samples from 11 hearts and the corresponding MRIs. On each image (*N* = 12), we measured along two distinct transmural trajectories yielding a total of 24 samples [Colour figure can be viewed at wileyonlinelibrary.com]

A visual comparison of histological sections against MRI suggested the spatial resolution of MRI was not sufficient to discriminate individual trabeculations (Figure 11a,b). Next, on 12 left ventricular transmural histological sections with very different degrees of trabeculation, we counted trabeculations along two trajectories per sample from epicardium to the inner‐most endocardium (Figure 11c). The same measurements were performed on MRI with high and low spatial resolution on the locations that the histological samples were taken from (Figure 11c,d). Significantly more trabeculations were detected at higher spatial resolutions (*p* < 0.001, Figure 11e), indicating that the number of trabeculations will likely be underestimated if counted on images from clinical MRI. Measuring on the same sections and along the same trajectories, we found that the T/C was slightly but significantly larger at higher spatial resolutions (*p* < 0.046, Figure 11f). This indicates that the compact layer thickness may be slightly over‐estimated if measured on images from clinical MRI.

### 3.6 The trabeculation of LV and RV are not correlated

**FIGURE 12:**
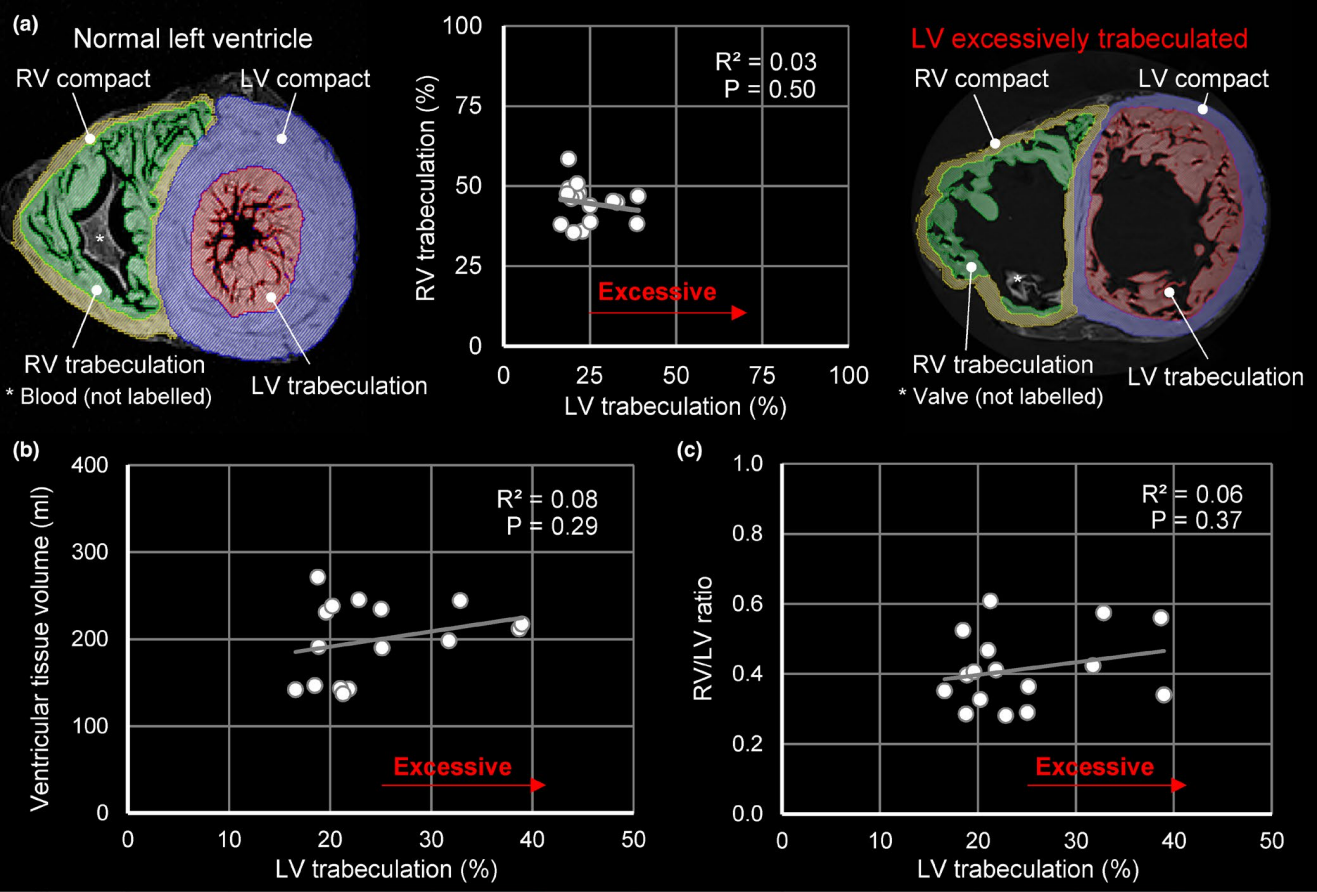
The proportion of trabeculation is not correlated between the ventricles. (a) The proportion of trabecular muscle in the left and right ventricle was not significantly correlated, despite a wide range of left ventricular trabeculation from normal (left‐hand image) to noncompacted (right‐hand image). (b–c) The proportion of left trabecular muscle was not correlated to total volume of the ventricles (b) or the relative volume of the RV to the LV (c) [Colour figure can be viewed at wileyonlinelibrary.com]

To test whether the extent of trabeculation of LV and RV was correlated, we first measured absolute volumes of LV and RV trabecular and compact layers (Table 3). In addition, the mean total ventricular tissue volume (approximately 200ml) and the ratio of right ventricular tissue volume to left ventricular tissue volume (approximately 0.4) were approximately normal (Dadgar et al., 1979). Note that there is a relatively large variation in left ventricular trabecular volume because of the inclusion of excessively trabeculated hearts. For the multiple linear regression analysis, we first tested with a Shapiro–Wilk test whether the distribution of the dependent variable (LV trabecular volume (ml)) deviated from normal and it did not (*p* = 0.346). We then correlated the volume of LV trabeculation to the volumes of LV compact, RV compact and RV trabeculation and found a significant correlation in the general model (adjusted *R* ^2^ = 0.343; *p* = 0.046). An effect of outliers was not evident, as all standardized residuals were between −3 and 3 (range −1.02 to 2.67). The RV trabecular volume was not significantly correlated to LV trabecular volume (*p* = 0.735), whereas there was a significant correlation to RV compact volume (Table 3). In addition, RV and LV trabecular volumes were also not significantly correlated if the volumes were expressed relative to total ventricular wall volume (Figure 12a). Since much of the variation in LV trabecular volume came from the excessively trabeculated hearts, we tested whether the LV trabecular proportion (%) was correlated to total ventricular volume and RV/LV ratio, and we found non‐significant correlations in both instances (Figure 12b,c). Thus, the excessively trabeculated hearts were not abnormal in total size nor in the size of their RV.

**TABLE 3:** Tissue volumes of the hearts that were investigated with MRI

	Average	STD	Maximum	Minimum	St. beta	*p* value
LV trabeculation (ml)	35.3	14.1	63.2	17.4	‐	‐
LV compact (ml)	107.3	30.4	171.4	67.2	0.261	0.248
RV trabeculation (ml)	25.2	6.5	39.9	14.1	0.086	0.735
RV compact (ml)	31.3	8.0	49.1	21.7	0.542	**0.047**
Ventricles (ml)	199.1	44.6	271.4	137.3		
RV/LV	0.41	0.11	0.61	0.28		

St. beta, standardized beta coefficient; STD, standard deviation.

## 4 DISCUSSION

We find higher spatial resolution impacts on the number of detectable trabeculations and on measurements of layer ratios such as T/C that are used to diagnose excessive trabeculation. This suggests that reported prevalence ranges of excessive trabeculation may be confounded by the historical and ongoing improvements of the spatial resolution of non‐invasive imaging. Our findings also suggest that improvements in spatial resolution may offer opportunities, such as the counting of trabeculations.

### 4.1 Prevalence of left ventricular excessive trabeculation

To find excessively trabeculated LVs, we screened 1112 autopsy hearts from two archives that are likely to be enriched in pathologies. Yet we identified five excessive trabeculated hearts only. Such low prevalence is consistent with previous pathology‐based investigations in which excessive trabeculation is considered rare (Burke et al., 2005; Dusek et al., 1975; Feldt et al., 1969; Finsterer & Zarrouk‐Mahjoub, 2013; Freedom et al., 2005; Grant & Regnier, 1926; Ursell, 2013). It is in contrast, however, to the prevalence based on non‐invasive imaging which is more than an order of magnitude greater, or approximately 10% (Ivanov et al., 2017; Kawel et al., 2012; Weir‐McCall et al., 2016; Zemrak et al., 2014). This suggests that pathology assessments do not assign diagnosis‐positivity to most hearts that are excessively trabeculated when *in vivo* non‐invasive imaging is used. This is unfortunate because pathology assessments impart validity to clinical diagnoses, in particular in cases of sudden cardiac death (Oechslin & Jenni, 2018). Compared with non‐invasive imaging, pathology assessments may have a reduced sensitivity in detecting excessive trabeculation because of several factors, such as variation in the planes of imaging or sectioning and a greater variation in the state of contraction (Gerger et al., 2013). At least in systole on clinical MRI, much of the trabecular layer changes in appearance to resemble compact wall (Grothoff et al., 2012). Whether these are the causal factors that could better be assessed by comparisons of hearts that were first assessed *in vivo* and then investigated *post*‐*mortem* by pathology assessments including *ex vivo* MRI assessments such as those performed here.

Left ventricles such as the ones shown in Figures 9, 10 are highly ambiguous with regards to diagnosis of excessive trabeculation. On the one hand, they are clearly not diagnosis‐positive, because the trabeculations comprise less than 25% of the LV wall and there is a well‐developed compact wall (T/C was less than 1.5 in segments 1–16 in both hearts). On the other hand, they are clearly excessively trabeculated because of either a highly trabecular outflow tract (Figure 9) or an excessive number of trabeculations (Figure 10). If a process of compaction reduces the number of trabeculations, including changing the ventricular septal crest from trabecular to compact, then these hearts can be considered ‘noncompacted’. Compaction and hence noncompaction, however, may only have a very limited role in determining the final proportion of trabecular to compact wall in human compared with differential growth rates of the trabecular and compact layer (Faber et al., 2021a). Also, genetically modified mice show that trabeculation can become so excessive that it vastly exceeds any normal stage (Rhee et al., 2018; Sandireddy et al., 2019) and the excessiveness of the trabeculation therefore cannot be explained by failed compaction alone.

### 4.2 Impact of spatial resolution

Pathology assessments of trabeculation in *post*‐*mortem* hearts may reveal features that are not easily captured by non‐invasive imaging of hearts *in vivo*. Trabeculation may be excessive in number by macroscopic inspection, even if they are difficult to count precisely (Gerger et al., 2013), and we show that greater spatial resolution in the range from clinical‐like MRI to histology yields a greater number of detected trabeculations. Since a greater‐than‐normal number of trabeculations must be a predicted outcome if ‘compaction’ has failed, the counting of trabeculations may be important to establish the aetiology of excessive trabeculation. Trabeculations can be found in parts that are normally not trabeculated such as the left ventricular outflow tract, although, surprisingly, the occurrence of outflow tract trabeculation appears to be independent of the setting of excessive trabeculation (Jacquier et al., 2010; Petersen et al., 2018; Stollberger & Finsterer, 2019; Thuny et al., 2010). Histology can reveal the fine spatial distribution of fibrosis, and our histology is in agreement with previous reports (Burke et al., 2005; Freedom et al., 2005; Jenni et al., 2001; Ursell, 2013) showing that fibrosis in excessively trabeculated ventricles is highly variable with no obviously distinctive patterns across cases. Also, histology allows for precise measurements of trabecular size. Size of the trabeculations is important in so far, it allows for the categorization of the trabeculations as being adult‐like (large) or embryonic‐like (less than 50µm wide and avascular), the latter of which would be expected if excessive trabeculation is the persistence of the embryonic ventricular design (Angelini et al., 1999; Freedom et al., 2005; Oechslin & Jenni, 2011). Although a great number of embryonically sized trabeculations have been reported in a few cases (Dusek et al., 1975; Steiner et al., 1996), we show here in agreement with previous studies (Angelini et al., 1999; Burke et al., 2005; Chin et al., 1990; Freedom et al., 2005; Jenni et al., 2001; Ursell, 2013; Val‐Bernal et al., 2010) that the trabeculations in most excessively trabeculated ventricles are much greater than embryonic trabeculations. We suggest, therefore, that these trabeculations should be considered distinct from the embryonic trabeculations (Jensen et al., 2016, 2017). In addition, such distinction should be made on the basis of histology and not with current clinical MRI due to insufficient spatial resolution.

### 4.3 Correlations of left and right ventricular trabeculation

Data from heart development show substantial similarity in the trabeculation of the LV and RV. Trabecular formation begins in the embryo in the early stages of chamber development under the influence of growth factors from the endocardium and transcription factors that are broadly expressed in the myocardium (Del Monte‐Nieto et al., 2018; Rhee et al., 2018; Sedmera et al., 2000; Sizarov et al., 2011; Stennard et al., 2003; Wilsbacher & McNally, 2016). Accordingly, trabecular volumes of both ventricles undergo a pronounced increase and they are therefore positively correlated in this period (Blausen et al., 1990; Faber et al., 2021b). Important developmental processes impact differently on the two ventricles, however. The LV derives from the linear heart tube and first heart field, whereas the RV is derived from the embryonic outflow tract and second heart field (Dyer & Kirby, 2009; Kelly et al., 2014). Trabeculation initiates later in the RV, and right ventricular trabeculations undergo greater thickening (Blausen et al., 1990; Crick et al., 1998; Faber et al., 2021b; Wenink, 1992). In addition, it has been shown that left ventricular trabeculation can grow such that infants become excessively trabeculated after birth (Stollberger et al., 2015) and women can become excessively trabeculated during pregnancy (Gati et al., 2014). In our assessment, the data on development of trabeculations do not indicate whether left and right ventricular trabeculation of the adult heart are correlated and our data suggest they are not.

To the best of our knowledge, only one previous study compared trabeculation of the two ventricles of normal adult hearts using *in vivo* imaging, and a moderate correlation (*R* ^2^ = 0.21) was found (Andre et al., 2015). Trabeculations in that study were measured as the area occupied by the trabeculations together with the intertrabecular recesses and trabeculations measured such has been used to diagnose excessive trabeculation (Jacquier et al., 2010). Later studies, however, have shown there is a greater sensitivity in detecting excessive trabeculation that associates with adverse outcomes when the intertrabecular recesses are excluded and only the trabeculations are measured (Grothoff et al., 2012; Macaione et al., 2021). In contrast to Andre et al. (2015), we did not find the left and right ventricular trabeculation to be correlated and we measured the volume of trabeculations only. In patient populations of excessive LV trabeculation, excessive trabeculation of the RV does occur, but it is much more frequent that only the LV is excessively trabeculated (Burke et al., 2005; Ichida et al., 1999; Nucifora et al., 2014). In excessive trabeculation of the RV, it is less clear how often the LV is excessively trabeculated, but cases of isolated excessive trabeculation of the RV do occur (Fazio et al., 2010; Ilyas et al., 2013; Montanarella et al., 2021). Such observations are consistent with at least some degree of independence between the LV and RV in the establishment of the final proportions of trabeculations.

## 5 CONCLUSION

Greater spatial resolution improves the detection of ventricular trabeculation of the human heart and this is most clearly seen in the number of counted trabeculations. One implication is that spatial resolution may affect the sensitivity of diagnostic measurements of excessive trabeculation. Improved imaging could allow for novel measurements such as counting of trabeculations, which is currently not possible in the clinic.

## AUTHOR CONTRIBUTIONS

Conceptualization of the study was by BJ; acquisition of data was by HCE, BFC, GS, ACvdWal and BJ; data analysis and interpretation were HCE and BJ; drafting of the manuscript was by HCE, BFC and BJ; critical revision of the manuscript was by SEP, MNS, R‐JO, VMC and BJ.

## Supporting information

Material S1

## Data Availability

The data that support the findings of this study are available from the corresponding author upon reasonable request.
